# Editorial: Reviews in networks in the brain system

**DOI:** 10.3389/fnetp.2024.1403698

**Published:** 2024-05-21

**Authors:** Cristina Masoller, Klaus Lehnertz, Marc Goodfellow, Dimitris Kugiumtzis, Michal Zochowski

**Affiliations:** ^1^ Department of Physics, Universitat Politecnica de Catalunya, Barcelona, Spain; ^2^ Department of Epileptology, University of Bonn Medical Centre, Bonn, Germany; ^3^ Helmholtz Institute for Radiation and Nuclear Physics, University of Bonn, Bonn, Germany; ^4^ nterdisciplinary Center for Complex Systems, University of Bonn, Bonn, Germany; ^5^ Living Systems Institute, University of Exeter, Exeter, United Kingdom; ^6^ Department of Electrical and Computer Engineering, Aristotle University of Thessaloniki, Thessaloniki, Greece; ^7^ Biophysics Program and Department of Physics, University of Michigan, Ann Arbor, MI, United States

**Keywords:** brain network, brain dynamics, intracranial EEG, imagination, consciousness, mathematical models, functional connectivity, structural connectivity

The human brain is a complex system whose temporally and spatially multiscale structure gives rise to an immense functionality, which can coexist with pathophysiologic functioning in case of diseased states. Many important advances in various fields of research critically continue to improve our understanding of the structure and (dys-)function of the complex network brain (Goodfellow et al., 2022) together with its interactions with other organ systems in the human body (Lehnertz et al., 2020; Ivanov, 2021). In addition to the neurosciences, lifesciences and humanities, we mention many branches of the natural, information, and data sciences, all of which are accompanied by an ever increasing technology. The exchange of knowledge and novel ideas in this highly interdisciplinary field of research is supported by high-quality scholarly review papers on key topics, such as those that make up this Research Topic of *Frontiers in Network Physiology–Networks in the Brain System* section. This article Research Topic–consisting of three review articles and one hypothesis and theory article–features contributions from leading experts that describe the state of the art, outlining recent developments and major accomplishments that have been achieved and that need to occur to move the field forward.

Mathematical models are crucial to understand the dynamics of the brain in physiological and pathophysiological conditions, at rest and during tasks. They allow us to think about underlying principles that may govern the production of changes in brain activity that are observed, for example, in electrophysiological recordings or behavioral phenotypes. State-switching dynamics are particularly important. They describe the transitioning of the brain between different types of activity, for example, different topographies of electrical fields in microstate analyses (Michel and Koenig, 2018), different arrangements of spatiotemporal patterns of fMRI in dynamic functional connectivity (Hutchison et al., 2013), or the emergence of seizures amongst otherwise healthy brain activity (Baier et al., 2012). In their article, Meyer-Ortmanns reviews a particular class of mathematical models that has the potential to help understand state-switching dynamics: heteroclinic networks. These are essentially dynamical systems that give rise to metastable sets connected by paths, such that the dynamics can switch between these sets over time. Hildegard Meyer-Ortmanns first motivates the importance of models that can capture key features of observed brain dynamics such as a balance between robustness and sensitivity to external stimuli, before providing an overview of the underlying mathematical ideas. Examples are then given of how heteroclinic networks can be used to model and understand important concepts such as segmentation and binding of different features, the processing of sequential information and non-Markovian properties of state switching in observations of microstate dynamics. There is also discussion of the design of heteroclinic network dynamics, the role of noise and external forcing and mechanisms of learning in heteroclinic networks.

There are thousands of published works of connectivity analysis on extracranial EEG but disproportionately less on intracranial EEG (iEEG). The Review by Novitskaya et al. discusses the limitations of connectivity analysis of iEEG, shifting the attention from a single brain region to interactions among distributed neuronal populations and brain areas. The review is equally shared to two approaches of brain connectivity regarding the state of iEEG, the one regarding resting state or under a task and the other regarding the response to stimulation called corticocortical evoked potentials (CCEP), typically single pulse electrical stimulation (SPES). Methodological challenges and issues in both approaches are discussed, which are different from these for the extracranial EEG. A number of works using SPES-based methods have established that cortical stimulation is an important tool for mapping connectivity between brain regions. In particular, characteristics of the CCEP were found to correlate to epileptogenic regions and seizure onset zone. A number of studies on interictal iEEG (no stimulation) suggest higher connectivity in brain areas located in the epileptogenic zone as compared to propagation zones and zones not involved in the seizure generation. The review concludes that the iEEG-based connectivity assessment has a promising application in mapping epileptogenic networks, and possibly in the near future developing neural biomarkers that have a supportive value for visual seizure onset zone search.

Understanding the coupling structure of interacting systems is an open challenge across disciplines, and a lot of efforts have been focused on reconstructing structural connectivity from observed data (see, e.g., Tirabassi et al. (2015); Rings et al. (2022) and references therein). The Mini Review by Rosenblum and Pikovsky discusses phase-based methods applied to interacting oscillatory systems. The key assumptions are i) that the oscillators’ signals can be described by appropriately defined phases and ii) the interaction strengths are sufficiently weak, and the system of *N* oscillators evolves in a *N*-dimensional torus. Then, structural information can be recovered after inferring an appropriated model for the dynamics of the phases. This approach is demonstrated with oscillators that have a smooth dynamics, and with oscillators that have a pulsed behavior. The authors first discuss different approaches to infer the phases of the oscillators and then show, for two o three smooth oscillators with undirected or directed links, how the phase model allows to infer the general forms of the coupling functions. High order terms in the coupling functions limit the precision of the model as the recovered connectivity differs from the real one. For pulsing oscillators, the timing of the pulses is used to define phases that grow linearly, from 0 to 2*π*, between two consecutive pulses, and these phases are in turn assimilated to a Winfree-type model, from which, the connectivity is recovered; however, discriminating weak coupling from no coupling remains a challenge.

The brain information processing can be divided into two forms of activity—explicit processing guided by ongoing sensory experiences that can be reported by the subject, and implicit, unconscious activity, based on skills and previous experience, that is thought to underlie imagination. The Hypothesis and Theory article by Fesce and Gatti discusses differential activation of brain networks involved in explicit (conscious) and implicit (unconscious) processing of motor imagery. To that extend, the authors compare and contrast cognitive pathways involved in motor execution, kinaesthetic motor imagery, visual motor imagery and action observation. They hypothesize that the observed differences in these pathways can be attributed to brain activity mediating access to conscious awareness, via selective attention mechanisms, and consisting of bottom-up processes linked to internal processing, and top-down processes associated with active processing of current fed sensory content. During unconscious activity the top-down control of selective attention would be weakened, and bottom-up component of attentional control would dominate in an unattended, unintentional way. This, the authors further argue, could happen via disinhibition of cortical pyramidal neurons leading to less discriminative activation of the cortex.

Though the collected papers are on brain function and brain networks, the concepts and methods can be extrapolated to interactions between the brain and other organ systems and to network physiology at large. We are confident that this article Research Topic will inspire, inform and provide direction and guidance to researchers in this cross-disciplinary field of research.
